# Black necrosis of the glans penis associated with calciphylaxis: A case report

**DOI:** 10.1097/MD.0000000000035609

**Published:** 2023-10-20

**Authors:** Youwei Yu, Yangxi Chen, Fan Yang, Qitai Song

**Affiliations:** a Department of Emergency Medicine, the Affiliated Hospital of Southwest Medical University, Luzhou, Sichuan Province, China.

**Keywords:** artery calcification, calciphylaxis, case report, chronic kidney disease, genital gangrene

## Abstract

**Rationale:**

Calciphylaxis, known as calcific uremic arteriolopathy, is a rare cause of dry gangrene. Despite an increase in the clinical recognition of demographic characteristics and risk factors associated with calciphylaxis, it remains a poorly understood disease with high mortality.

**Patient concerns and diagnoses:**

We present a 45-year-old man, who was diagnosed with calciphylaxis disease, with a history of diabetes mellitus, end-stage renal disease and cirrhosis with a half-month evolution of painful dry gangrene on his glans penis and scrotum. The patient also presented with gangrene of fingers.

**Interventions and outcomes:**

The patient and his family opted for palliative care. However, he died eventually.

**Lessons:**

This case contributed to the current understanding of calciphylaxis. Since no standard treatment is available and the prognosis remained poor, early, and accurate diagnosis of calciphylaxis is important. We here report the current case and provide data for the diagnosis and treatment of this kind of disease.

## 1. Introduction

Calciphylaxis is a rare, life-threatening syndrome of vascular calcification characterized by occlusion of microvessels in the subcutaneous adipose tissue and dermis that results in intensely painful, ischemic skin lesions.^[1]^ Risk factors for calciphylaxis are obesity, diabetes mellitus, female sex, and end-stage renal disease depending on dialysis.^[2]^ It is a devastating condition with important systemic ramifications.^[3]^ The disorder typically affects patients with end-stage renal disease,^[4]^ a population with a high prevalence of extraskeletal calcifications. Anogenital, including penile and scrotum, involvement by calciphylaxis is rare and portends a poor prognosis. Patients may delay seeking medical advice in the case of genital and scrotum lesions and as a result of this, patients will have a poor prognosis. We report herein a patient with calciphylaxis who presented with a painful dry gangrene of the penis and scrotum. Penile involvement of calciphylaxis is rare. This results in exquisitely painful and slow healing wounds that portend exceptionally high morbidity and mortality.^[5]^ This case further highlights the fact that calciphylaxis is a systemic vascular disease with poor prognosis.

## 2. Case presentation

**Figure 1.**
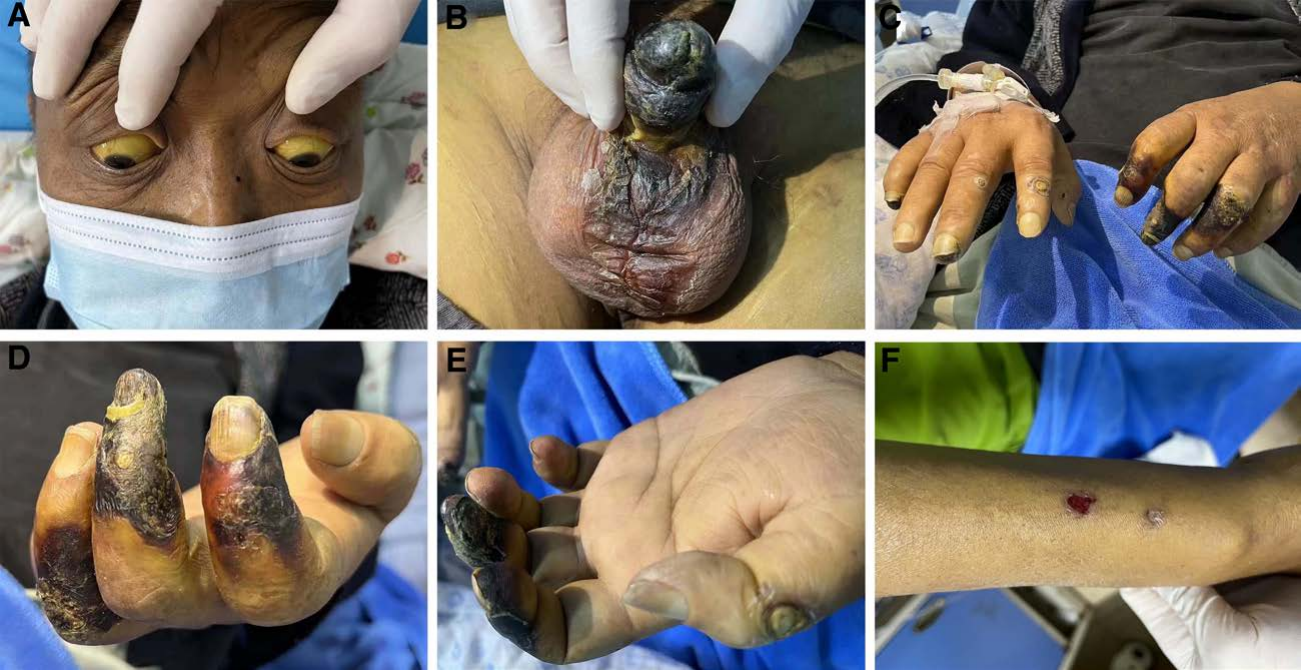
(A) Yellow skin and sclera. (B) Entirety of the glans penis with black (dry) necrotic lesion and black (dry) necrotic lesion of the scrotum. (C–E) Acral calciphylaxis. Fingers with a stellate ulceration with overlying black eschar and surrounding violaceous patch. (F) Calciphylaxis. Forearm radial side with focal erosion.

A 45-year-old man with a history of type II diabetes mellitus, cirrhosis, heart failure and arteriosclerotic occlusive disease presented to the emergency department. He also had chronic kidney disease stage 5 and had been on hemodialysis for over 1 year. He was admitted to the hospital for yellow skin and sclera of his general condition (Fig. 1A), accompanied by loss of appetite and general malaise that had started 7 months prior to admission. During hospitalization, the patient complained of severe pain of his penis, scrotum and fingers, especially the penis. The initial lesions rapidly progress to malodorous ulcers with black eschars. Physical examination revealed black (dry) necrotic lesion of the penile and scrotum suggestive of deep tissue necrosis (Fig. 1B). The patient presented concomitantly with painful yellow and black (dry) necrotic lesions of fingers, which had been spontaneously deteriorating (Fig. 1C–E). Physical examination also revealed forearm radial side with focal erosion (Fig. 1F). What’s more, the radial artery, ulnar artery, brachial artery, and bilateral dorsal pedar artery of patient are not palpable.

The patient’s laboratory workup revealed leukocytosis of 23.10 × 10^9^/L with neutrophil predominance, procalcitonin of 2.13 ng/mL (normal range 0–0.05 ng/mL), hemoglobin of 74 g/L, NT-pro-brain natriuretic peptide of 14935 ng/L(normal range 0–88 ng/L), albumin of 29.2 g/L, total bilirubin of 324.5 µmol/L, direct bilirubin of 286.6 µmol/L, total bile acid of 150.8 µmol/L, creatinine of 369.6 µmol/L(normal range 57–97 mmol/L), urea of 13.08 mmol/L(normal range 3.1–8.0 mmol/L), calcium of 2.4 mmol/L, and glomerular filtration rate of 16.3 mL/min. His blood analysis demonstrated normal parathyroid hormone.

**Figure 2.**
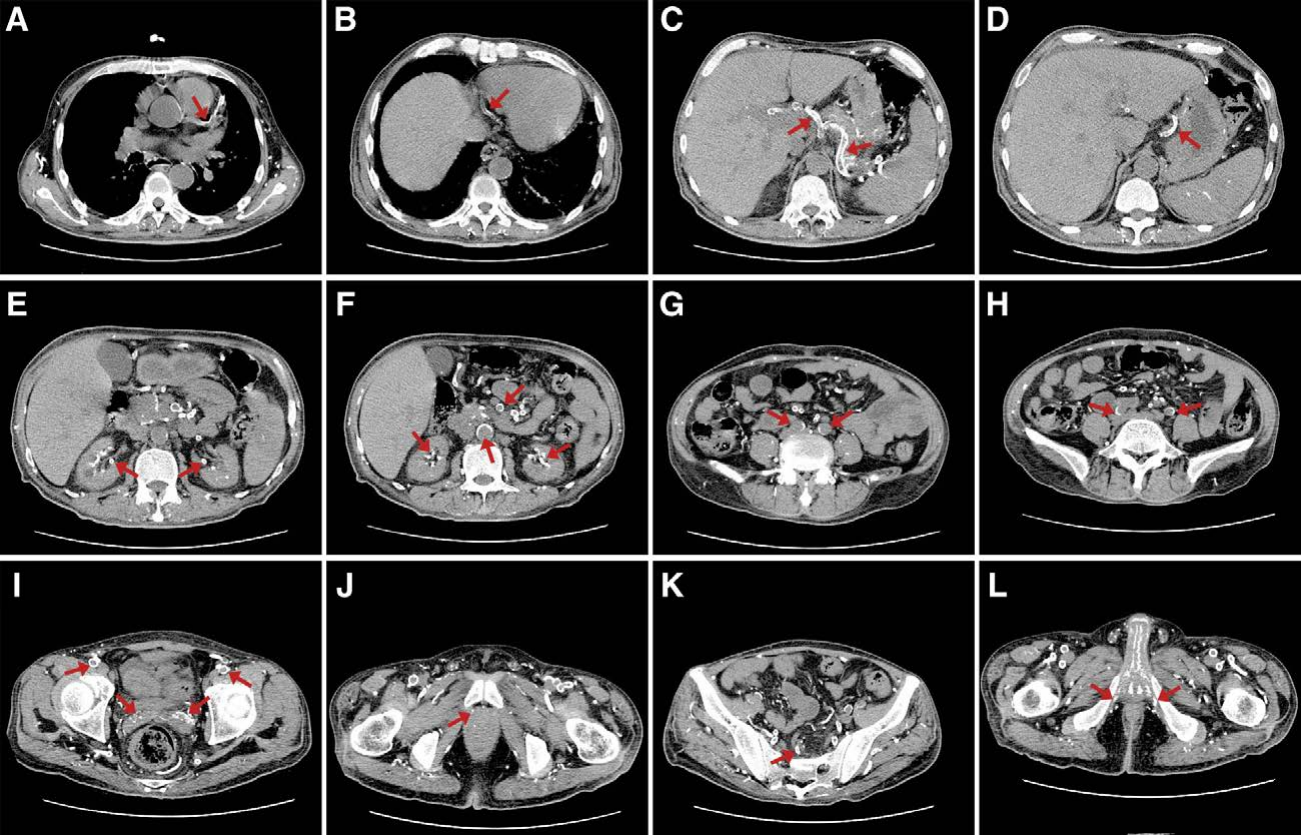
(A) Calcification of left coronary artery. (B) Calcification of right coronary artery. (C) Calcification of common hepatic artery and splenic artery. (D) Calcification of left gastric artery. (E) Calcification of renal vessels. (F) Calcification of abdominal aorta, superior mesenteric artery and renal tissues. (G) Calcification of common iliac arteries. (H) Calcification of external iliac arteries. (I) Calcification of femoral arteries and seminal vesicle. (J) Calcification of prostate. (K) Calcification of rectal vessel. (L) Calcification of internal pudendal arteries.

In November, 2021, abdomen routine scan of patient showed calcification of the splenic artery. Within 1 year, the extent of vascular calcifications of patient deteriorated. Recent chest routine scan shows that calcification of coronary arteries, aorta vessel walls and part of aortic arch vessel walls. Plain computed tomography of abdomen and pelvis shows widespread calcification of renal arteries, abdominal aorta, hepatic artery, common iliac artery, internal and external iliac arteries and their branches, and pudendal artery with significant calcified plaques, causing significant stenosis. Especially, diffuse aortic calcification and dense calcification of internal and external iliac arteries. In addition to vascular calcification, calcification involves other organs of patient, such as thyroid gland, prostate, epididymis, corpora cavernos, and seminal vesicles (Fig. 2).

Hospital multidisciplinary group hold a consultation on this patient. The differential of tissue necrosis is broad, and identifying calciphylaxis requires an adroit understanding of the risk factors and physical signs. According to the patients’ condition, the differential diagnosis of the penile ulcer included calciphylaxis, diabetic necrobiosis, irritative contact dermatitis, vasculitis, vascular embolism, thrombosis, and Fournier gangrene. The patient’s condition was complicated by cirrhosis, intractable pain of the penis, and fingers.

The group holds the view that systemic vascular lesions of the patient with multiple underlying diseases are caused by calciphylaxis. The course of patient had been deteriorated rapidly. At present, it is challenged to make a perfect treatment for patients with abnormal functions of multiple organs, black gangrene of both fingers and external genitalia, and co-infection. The patient and his family opted for palliative care and treated with meropenem, prostaglandin E1, ursodeoxycholic acid capsules, injection of insulin, torasemide capsules, nutritional support, and three-times-a-week hemodialysis. We sought advice from andrology center of our hospital. We managed the penile lesion conservatively. The lesion was kept dry and disinfection. His calcium and renal function were monitored, while also undergoing his normal hemodialysis regimen.

The patient and his family refused to continue the treatment and asked to discharge 3 weeks later with personal reasons. The patient died at home 25 days later.

## 3. Discussion

Calciphylaxis is an uncommon but devastating disorder characterized by vascular calcification and subsequent cutaneous tissue necrosis. Calciphylaxis had been considered to primarily affect patients with end-stage renal disease requiring dialysis; however, it is can also affect patients with normal kidney function.^[6]^ Its pathogenesis remains poorly understood. According to lesion localization, calciphylaxis is classified as proximal/central, involving central subcutaneous adipose tissue areas, or distal/peripheral, when it is restricted to peripheral sites with limited adipose tissue. Six-month mortality and 1-year mortality of patients with calciphylaxis was 37.2% and 44.1%, respectively. Patients with nephrogenic calciphylaxis had worse survival than those with nonnephrogenic calciphylaxis. Age and end-stage renal disease were risk factors associated with 1-year mortality.

Penile involvement is a rare distal manifestation of calciphylaxis; it manifests with black eschars and necrosis mimicking a wound. Moreover, treatment for calciphylaxis remains challenging as there are no randomized controlled trials to evaluate the efficacies of different treatment options. Prevention of soft tissue and vascular calcification depends upon early aggressive medical management. Patients with penile calciphylaxis were more likely to have hyperparathyroidism and an elevated the parathyroid hormone level at the time of diagnosis compared with controls. Uncontrolled hyperparathyroidism is historically considered to be the cause of calciphylaxis, and early aggressive medical management of hyperparathyroidism is suggested,^[7]^ however, recent data suggest that uncontrolled hyperparathyroidism is not the key determinant of calciphylaxis.^[8]^ The normal parathyroid hormone level of our case patient validated this view.

As there are no conclusive serologic, radiographic or visual signs that this disease is manifesting, the diagnosis of this condition can be complicated. Even in patients with a high clinical suspicion, calciphylaxis has traditionally been difficult to confirm. When confirming a high clinical suspicion for calciphylaxis, imaging and tissue biopsy are useful adjunct diagnostic modality. Because of lack of subcutis or nonspecific calcification patterns, histopathology was equivocal about imaging supported calciphylaxis cases. While the classic recommendation of an incisional biopsy may increase diagnostic yield, it is not always practical when weighed against patient tolerability, infection risk and impaired wound healing.^[9]^ When calciphylaxis involves the penis, biopsy specimen may even be contraindicated.

Our case suggests that calciphylaxis should be considered while evaluating skin lesions in patients with predisposing conditions in the condition of end-stage kidney disease and cirrhosis. There is no standardized local or systemic treatment exists, so the treatment of genital calciphylaxis is challenging.

The management should be discussed in a multidisciplinary group. If the lesions progress to gangrene and sepsis, surgical intervention may be needed to be taken into consideration. However, after surgical intervention, a pathergic reaction can also cause progression of necrosis. Some cases reported that treatment with 25 g of intravenous sodium thiosulphate with dialysis 3 times weekly for 6 months, is able to resolve the calciphylaxis.^[10]^ Unfortunately, our patient was not given this for his treatment.

Therefore, we should consider the patient’s preference and his life expectancy. Palliative care services, which are specialized in symptom management, may be beneficial in such patients, who are suffering from this painful and debilitating condition. Our patient developed simultaneously 2 manifestations of calciphylaxis, penile and fingers involvement, which is rare in clinical cases. All in all, early recognition of this disease is essential for leading to rapid treatment initiation and is an important step in improving outcomes of patient. One important point is that pain management and wound care are essential and we should try our best to maintain the quality of life of the patient.

## 4. Conclusion

Calciphylaxis should be considered as a possible cause of penile necrotic lesions. Penile involvement in calciphylaxis is very rare and manifests as a black (dry) necrotic lesion and the survival was significantly worse. We reported a case of penile gangrene caused by calciphylaxis associated with chronic kidney disease, type II diabetes mellitus, cirrhosis, and co-infection with long history with smoking. In typical cases, clinical presentation, physical examination and imaging can be used to aid diagnosis and at the same time avoiding risks associated with biopsy. Since no standard treatment is available, management should be based on a multidisciplinary approach.

## Author contributions

**Conceptualization:** Youwei Yu.

**Formal analysis:** Youwei Yu.

**Investigation:** Youwei Yu.

**Methodology:** Yangxi Chen.

**Resources:** Fan Yang.

**Supervision:** Fan Yang.

**Visualization:** Yangxi Chen.

**Writing – original draft:** Youwei Yu.

**Writing – review & editing:** Qitai Song.
